# Extramedullary relapse of acute myeloid leukemia in brachial plexus after allogeneic stem cell transplantation: a case report

**DOI:** 10.1186/s12883-022-02768-1

**Published:** 2022-07-01

**Authors:** Shogo Shirota, Daisuke Katoh, Yoshimitsu Shimomura, Yukihiro Imai, Takayuki Ishikawa

**Affiliations:** 1 Department of General Internal Medicine, Kobe City Medical Center General Hospital, 2-1-1 Minatojima-minamimachi, Chuo-ku, Kobe, Japan; 2 Department of Hematology, Kobe City Medical Center General Hospital, 2-1-1 Minatojima-minamimachi, Chuo-ku, Kobe, Japan; 3 Department of Pathology, Kobe City Medical Center General Hospital, 2-1-1 Minatojima-minamimachi, Chuo-ku, Kobe, Japan

**Keywords:** Extramedullary relapse, Brachial nerve, Acute myeloid leukemia, Allogeneic stem cell transplantation

## Abstract

**Background:**

Allogeneic hematopoietic stem cell transplantation is a potentially curative treatment for acute myeloid leukemia. However, extramedullary relapse of acute myeloid leukemia can occur after hematopoietic stem cell transplantation, causing treatment failure. Extramedullary relapse rarely involves the peripheral nerves, and it is not influenced by the effect of the graft on leukemia.

**Case presentation:**

We report a case of extramedullary relapse of acute myeloid leukemia in the brachial plexus of a 41-year-old woman treated with allogeneic hematopoietic stem cell transplantation (HSCT). Complete hematological remission was confirmed by bone marrow examination 1 month after HSCT, and she developed no major complications immediately after HSCT. The immunosuppressant was discontinued 5 months later. However, 2 weeks after immunosuppressant withdrawal, the patient developed left arm pain and paresthesia, with subsequent development of a mass in the left brachial plexus. She was initially diagnosed with brachial plexus neuropathy because of concomitant graft-versus-host disease. Despite the administration of immunosuppressive agents, the mass continued to enlarge. The biopsy of the lesion revealed leukemic relapse. Thus, the patient was diagnosed with extramedullary relapse and underwent radiotherapy, resulting in tumor shrinkage.

**Conclusion:**

Extramedullary relapse should be considered a differential diagnosis in post-transplant patients with leukemia presenting with paresthesia.

## Background

Allogeneic hematopoietic stem cell transplantation (HSCT) is a potentially curative treatment for acute myeloid leukemia (AML). However, relapses can occur after HSCT, causing treatment failure. Extramedullary relapse (EMR) of AML with or without bone marrow relapse sometimes occurs after HSCT, and its prognosis is poor. Previous studies have shown that the incidence of EMR after HSCT was 2.4%–10.2% [1–3]. The common sites of involvement are the skin, soft tissue, lymph nodes, bone, and central nervous system [1–3]. However, EMR rarely involves the peripheral nerves, and it is not influenced by the effect of the graft on leukemia [3, 4]. There have been few investigations on the occurrence of EMR in the brachial plexus. We report a case of EMR that initially involved the brachial plexus in a patient who developed chronic graft-versus-host disease (cGVHD) after successful allogeneic HSCT and radiotherapy for AML.

## Case presentation

**Fig. 1:**
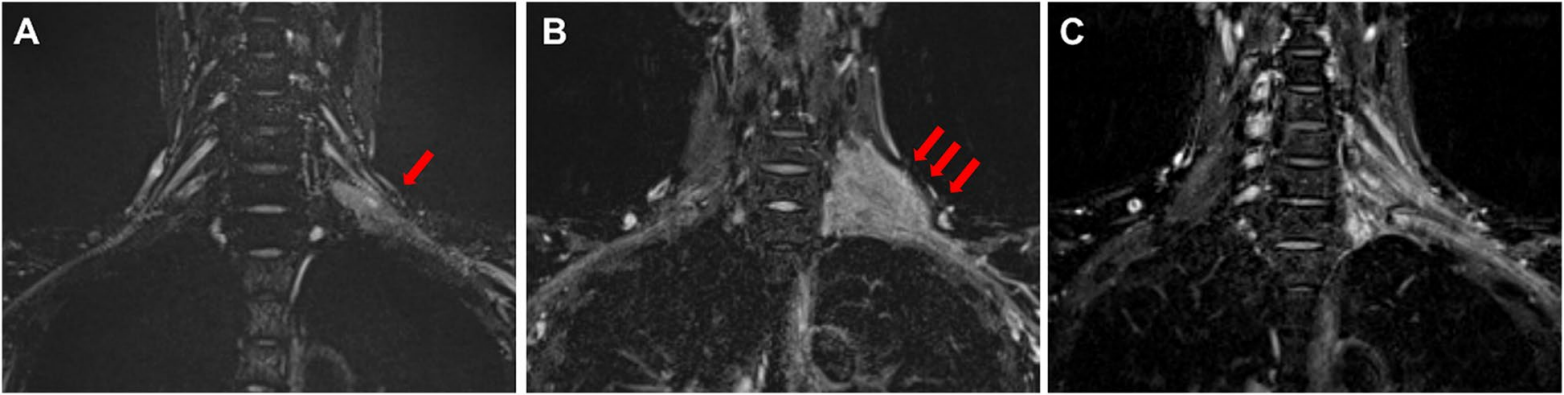
Magnetic resonance imaging of the brachial plexus. **A** Coronal short inversion time inversion recovery (STIR)-weighted magnetic resonance imaging (MRI) with contrast performed 38 days after the onsent of pain in her extremities shows hyperintense signals of the brachial plexus swelling (arrow) with enhancement at diagnosis. **B** Coronal STIR-weighted MRI performed 3 months after pain onset shows enlarged mass lesion. **C** Coronal STIR-weighted MRI performed 40 days after the completion of radiation therapy shows significant tumor shrinkage. MRI: magnetic resonance imaging, STIR: short inversion time inversion recovery

**Fig. 2:**
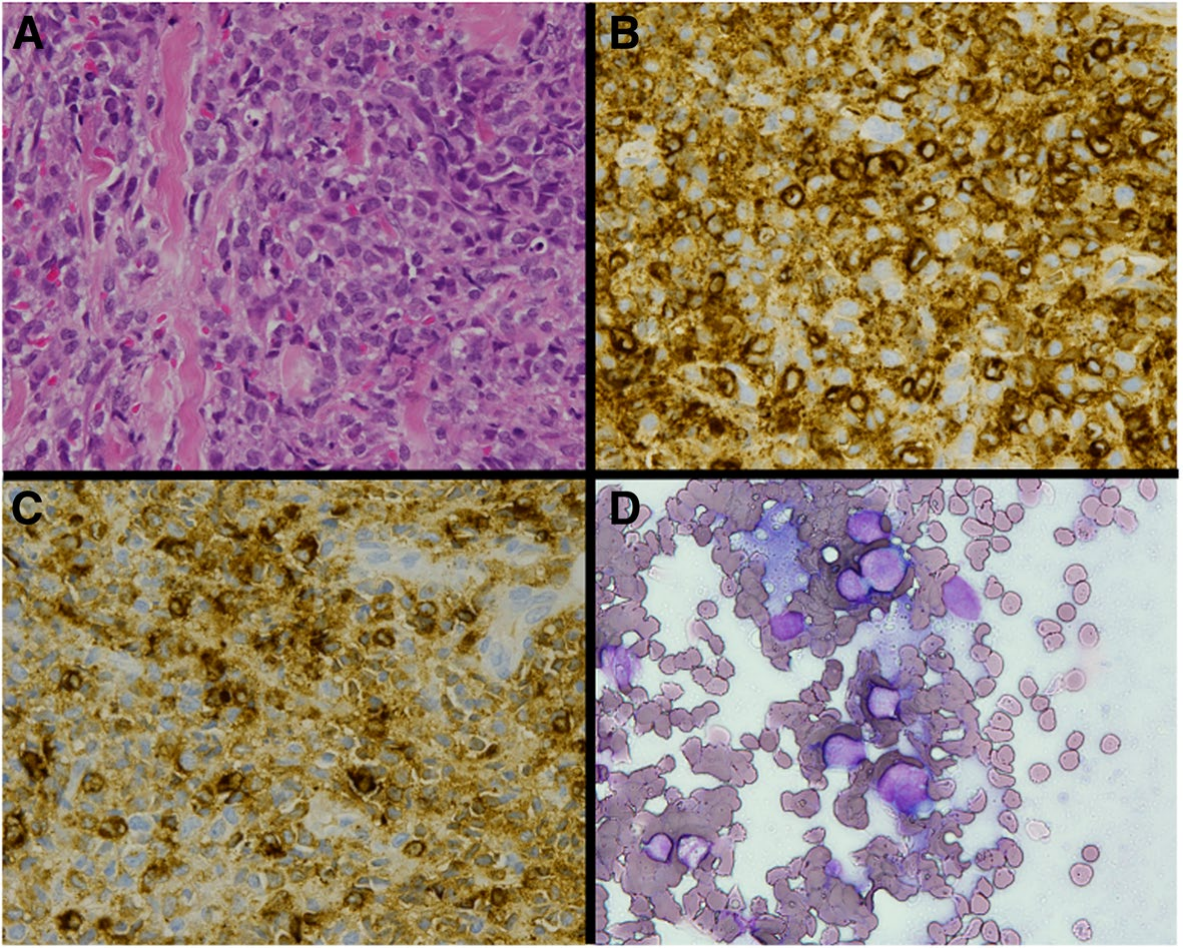
Biopsy of brachial plexus. **A** Hematoxylin and eosin staining of the brachial plexus-biopsy specimen shows a diffuse infiltrate composed of monocytoid cells with large, prominent nucleoli (magnification: ×40). **B**, **C** Immunohistochemical stains for myeloperoxidase and lysozyme are positive in the abnormal monocytoid cells (magnification: ×40). **D** May-Giemsa staining of the brachial plexus-biopsy specimen shows abnormally large cells with a high nuclear/cytoplasmic ratio (magnification: ×60)

A 41-year-old woman was diagnosed with AML according to the French-American-British Classification M5 with an FMS-like tyrosine kinase 3 (FLT3) mutation. She received two cycles of induction chemotherapy, but complete remission was not achieved. The patient underwent peripheral blood stem cell transplantation from her matched sibling. The conditioning regimen consisted of cyclophosphamide (30 mg/kg/day for 5 days) and total body irradiation (3 Gy for 4 days). The graft-versus-host disease (GVHD) prophylaxis consisted of tacrolimus and short-term methotrexate. Complete hematological remission was confirmed by bone marrow examination 1 month after HSCT. She developed no major complications immediately after HSCT, and the immunosuppressant was discontinued 5 months later. However, pain in the bilateral upper extremities was noted 2 weeks after the withdrawal of immunosuppressant. In addition, she presented with lichen planus-like lesions in the mouth and a corneal ulcer associated with dry eyes. Her symptoms were persistent at the follow-up visit 10 days later, and laboratory examination revealed elevated liver enzymes. She was admitted to our hospital with a working diagnosis of cGVHD. The pain in her bilateral extremities gradually transitioned to localized paresthesia in her left forearm and hand during the following 2 weeks. She was examined by a neurologist, who observed normal sensations and strength in the extremities. Her paresthesia was localized to the left forearm and hand, predominantly in the first to third fingers. There was no visible or palpable mass in the neck. Her deep tendon reflexes were normal, and a nerve conduction study for the left upper extremity showed unremarkable findings. At this point, our team and the neurologist established differential diagnoses of myofascitis, cervical spondylotic myelopathy, idiopathic brachial plexopathy, GVHD-associated brachial plexopathy, and EMR of AML. Contrast-enhanced magnetic resonance imaging (MRI) performed 38 days after onset of pain in her extremities revealed a perineural enhanced swelling lesion in the left brachial plexus (Fig. 1A). Bone marrow examination revealed no signs of relapse. She was diagnosed with cGVHD-associated neuropathy based on the characteristic oral and ocular symptoms and was started on prednisone and tacrolimus. Upon receiving immunosuppressants, her symptoms improved, except for the upper extremity paresthesia. Because neuropathy was not histopathologically confirmed, we closely monitored her condition, and follow-up MRI was performed 3 months after pain onset. Enlarged mass lesion in the left brachial plexus was observed (Fig. 1B). In addition, the mass was visible and palpable in the left supraclavicular region. At this point, the lesion was suspected to be an EMR of AML, with no association with cGVHD. Thus, a biopsy of the left brachial plexus was taken, and it revealed abnormal monocytoid cell infiltration (Fig. 2A-D). After confirming the diagnosis of EMR of AML, radiation therapy for the mass in the brachial plexus was initiated, leading to tumor shrinkage that was observed on MRI performed 40 days after the completion of radiotherapy (Fig. 1C). Two months after the completion of radiation therapy, hematological relapse occurred, and the patient received an FLT3 inhibitor. The patient remained in complete remission for the next 4 years.

## Discussion and conclusion

We reported the case of an AML patient who underwent HSCT and developed brachial plexus neuropathy due to leukemic infiltration and simultaneous cGVHD of the other organs. The biopsy of the brachial plexus aided in the accurate diagnosis of this case. The patient subsequently underwent radiotherapy, resulting in symptom resolution and tumor shrinkage. EMR rarely involves the peripheral nerves, but it should be considered in patients with a history of AML. In the present case, the patient concurrently developed cGVHD, which was a characteristic finding of the disease. This case suggested the importance of taking a biopsy in patients with cGVHD. EMR should be included in the differential diagnosis even when cGVHD occurs simultaneously because the protective effect of graft-versus-leukemia (GVL) against EMR is minimal.

EMR of AML occurs in approximately 10% of patients after HSCT [3]. There have been few reports on EMR in the brachial plexus. Only six cases of brachial plexus neuropathy due to leukemic infiltration have been reported. Three cases of EMR in the brachial plexus without HSCT were diagnosed clinically due to systemic relapse [5–7]. In addition, three previous case reports involved cases of EMR in the brachial plexus in patients with AML who underwent HSCT [8, 9]. Two of these cases were definitively diagnosed via biopsy and successfully treated with chemotherapy. In another case report, a 37-year-old patient with AML developed EMR after HSCT. He was initially misdiagnosed to have an immune response because a biopsy was not performed. The inaccurate diagnosis resulted in tumor-related death [8]. In our case, the patient developed isolated EMR plexus after HSCT, which was diagnosed via biopsy and successfully treated with radiotherapy and chemotherapy. Isolated EMR should be included in the differential diagnosis in patients with brachial plexus neuropathy. Moreover, a biopsy should be performed to achieve an accurate diagnosis.

The GVL effect did not protect against EMR [3, 4]. A single-center retrospective analysis demonstrated that cGVHD was associated with a lower incidence of overall relapse. However, it was not associated with EMR, suggesting that the GVL effect did not extend to the extramedullary sites [3]. Previous case reports of EMR in the brachial plexus revealed that GVHD occurred before or after the relapse [8–10]. Our patient had concurrent development of cGVHD and EMR in the brachial plexus. Concurrent development of EMR and GVHD poses a diagnostic challenge at the early stages of the disease [10]. When EMR and GVHD occur concomitantly, it is difficult to determine whether the neurological symptoms are caused by leukemic infiltration or immune-mediated inflammation, such as GVHD or idiopathic brachial neuritis [11]. Immune-mediated neuropathies such as cGVHD-associated Guillain–Barré syndrome generally present with symmetrical symptoms; however, in one reported case, the patient presented with asymmetrical cGVHD-associated neurological symptoms, as observed in the present case, although this condition is very rare [12, 13]. Therefore, a biopsy or brachial plexus mass excision is necessary because other diagnostic tools, including imaging findings, sometimes lead to an inaccurate or unclear diagnosis. Moreover, the management plans for EMR and immune-mediated inflammation are different [10]. In our case, the brachial plexus mass enlarged despite treatment for GVHD, and systemic relapse did not occur. Therefore, a diagnostic biopsy was performed, and the patient was successfully diagnosed with and treated for EMR.

In conclusion, the present case demonstrated an isolated brachial plexus relapse of AML that occurred simultaneously with cGVHD after HSCT. Leukemic infiltration and GVHD should be included in the differential diagnosis for patients with AML presenting with neuropathy. Taking a biopsy of the lesion is crucial to achieve an accurate diagnosis and provide proper treatment.

## Data Availability

All data analyzed during this study are included in this manuscript.

## Abbreviations

EMR: Extramedullary relapse
HSCT: Hematopoietic stem cell transplantation
AML: Acute myeloid leukemia
FLT3: FMS-like tyrosine kinase 3
cGVHD: Chronic graft-versus-host disease
GVHD: Graft-versus-host disease
MRI: Magnetic resonance imaging
GVL: Graft-versus-leukemia
